# Intelligence quotient decline following frequent or dependent cannabis use in youth: a systematic review and meta-analysis of longitudinal studies

**DOI:** 10.1017/S0033291720005036

**Published:** 2021-01

**Authors:** Emmet Power, Sophie Sabherwal, Colm Healy, Aisling O’ Neill, David Cotter, Mary Cannon

**Affiliations:** 1 Department of Psychiatry, Royal College of Surgeons in Ireland, Education and Research Centre, Beaumont Hospital, Dublin 9, Ireland; 2 Trinity College Institute of Neuroscience, Trinity College Dublin, Dublin 2, Ireland

**Keywords:** Cannabis, IQ, Neurodevelopment, Youth Mental Health, Meta-analysis, Longitudinal

## Abstract

Previous systematic reviews and meta-analyses of cross-sectional data assessing the effect of cannabis on cognitive functioning and intelligence show inconsistent results. We hypothesized that frequent and dependent cannabis use in youth would be associated with Intelligence Quotient (IQ) decline. This study is a systematic review and meta-analysis. We searched Embase, PubMed and PsychInfo from inception to 24 January 2020. We included studies with non-treatment seeking samples and pre- and post-exposure measures of IQ. We requested data from authors if summary data was not available from published work. We preregistered our review with PROSPERO (ID no. CRD42019125624). We found seven cohort studies including 808 cases and 5308 controls. We found a significant effect for the association between frequent or dependent cannabis use in youth and IQ change, Cohen's *d* = −0.132 (95% CI −0.198 to −0.066) *p* < 0.001. Statistical heterogeneity between studies was also low at *I*^2^ = 0.2%. Study quality was moderate to high. This translates to an average decline of approximately 2 IQ points following exposure to cannabis in youth. Future studies should have longer periods of follow up to assess the magnitude of developmental impact.

## Introduction

Cannabis is the most frequently used illicit substance worldwide, with the prevalence of lifetime cannabis use highest in young people (Degenhardt et al., 2013). Cannabis use in adolescence is consistently associated with poorer mental health outcomes including increased risk of mood disorders, self-harm and suicidality (Gobbi et al., 2019; Twomey, 2017). Cannabis use is also associated with markedly poorer psychosocial outcomes across the lifespan in diverse indices such as educational attainment, employment, relationships, welfare dependency, risk of motor accidents, social mobility and income (Fergusson, Horwood, & Beautrais, 2003; Fergusson, Lynskey, & Horwood, 1996; Hall, 2015). There is strong evidence demonstrating an association between cannabis and psychotic disorders, particularly frequent use of high tetrahydrocannabinol potency cannabis (Di Forti et al., 2019). Cannabis use has been estimated to be associated with approximately 12 and 15 excess life-years lost in women and men, respectively, in Danish register data (Weye et al., 2020). Earlier initiation of cannabis use and frequent cannabis use in adolescence are risk factors for later cannabis dependency (Leung, Chan, Hides, & Hall, 2020). Only a minority of those who have used cannabis more than five times in adolescence remit from use in mid-life, indicating the persistence of cannabis use (Perkonigg et al., 2008). One in three youth who use cannabis weekly or more frequently is cannabis-dependent (Leung et al., 2020). The legalisation of cannabis and a decreasing perception of harm in adolescent and young adult populations is likely to lead to increased use, particularly in vulnerable populations, resulting in negative public mental health consequences. (Mauro et al., 2019).

Cannabis use during youth is of particular concern, as the developing brain may be particularly susceptible to harm during this period (Lubman, Cheetham, & Yücel, 2015). A New Zealand cohort study has shown that persistent cannabis dependency from adolescence to midlife has previously been associated with a clinically significant eight-point decline in Intelligence Quotient (IQ) (Meier et al., 2012). The long-term effect of cannabis on intelligence is under-research. A recent study has found that even minimal incidental use of cannabis in adolescence is associated with morphological brain volume changes (Orr et al., 2019). A meta-analysis of cross-sectional MRI studies found replicated evidence of reduced grey matter in the CB1R rich areas of the hippocampus and the amygdala associated with cannabis use (Rocchetti et al., 2013).

Previous meta-analyses show inconsistent and heterogeneous findings for both global and specific cognitive domains relating to cannabis use. Two reviews found some evidence for deficits in attention, executive functioning, memory and learning, motor function deficit and verbal cognition (Ganzer, Broning, Kraft, Sack, & Thomasius, 2016; Grant, Gonzalez, Carey, Natarajan, & Wolfson, 2003). Two further meta-analytic studies found multidomain and overall cognitive deficits associated with cannabis use, however, reported that the results could be attributed to residual (i.e. may be related to recent use) rather than chronic effects (Schreiner & Dunn, 2012; Scott et al., 2018). The majority of studies included in these reviews have been case-control or cross-sectional studies generally containing small samples that may not be representative of the general population. Representative longitudinal cohort studies accounting for pre-cannabis exposure IQ may better inform whether frequent or dependent cannabis use in youth has a deleterious effect on IQ over time at a population level. This is to our knowledge the first meta-analysis of longitudinal IQ change in relation to cannabis use in adolescence.

The primary aim of this study is to quantitatively synthesize the available literature examining the longitudinal association between frequent/dependent cannabis use and IQ change from pre-exposure baseline in young people. We had a number of exploratory analyses. We explored whether we could disentangle the effects of chronic *v.* residual effects from the available longitudinal literature. Chronic effects are defined as effects lasting beyond a period of 28 days from last use and residual effects are effects lasting up to 28 days from last use (Pope et al., 2003). We also explored whether frequent/dependent cannabis use was associated with verbal and performance IQ decline, and lower baseline full scale, verbal and performance IQ.

## Methods

We preregistered our review with PROSPERO (ID no. CRD42019125624). We searched Embase, PubMed and PsychInfo from inception to 24 January 2019. We developed our search strategy through an iterative process with an information specialist to maximise the number of potential articles available for screening (see supplementary details for full search summary). Two authors independently screened articles by title and abstract to identify articles suitable for full-text review, following this, two authors screened articles by full text for inclusion in systematic review and meta-analysis.

We included prospective cohort studies of non-treatment seeking youth from samples recruited from the community with a baseline measurement of IQ prior to participants initiating cannabis use. We specified that the onset of cannabis use should have occurred at or before age 26. We specified that participants should have both a baseline and follow-up measure of IQ. We specified that studies should have at least a verbal and performance subtest of IQ allowing construction of a short form full-scale IQ composite measure. We considered articles or conference abstracts published in English. We defined our cannabis exposure as at minimum weekly use for 6 months and/or >25 reported lifetime uses and/ or diagnosis of cannabis dependency. The rationale for these thresholds was that approximately 1/3rd of weekly or greater adolescent cannabis users are cannabis-dependent and that studies would vary in how they measured cannabis use (i.e. some would measure lifetime use, some would define frequency, some would use diagnostic assessments) (Leung et al., 2020). We defined the control group as having used no or minimal cannabis (i.e. <5 lifetime uses). Where studies presented multiple groups i.e. frequent/dependent former and current users corresponding to chronic effects and residual effects respectively, we decided *a priori* to include them as one group in the main analysis, and attempt to separate them in exploratory analyses.

Two authors (EP, SS) using a pre-specified template extracted data independently. Disagreements were resolved with consensus through discussion. Where estimation of effect size was not possible with the available data or whereby the analytic strategy of the source data did not meet our inclusion criteria, we contacted authors to provide additional data/clarification. Two authors calculated effect sizes (EP, CM) agreement was 100%. We used WebPlotDigitizer to extract information from figures (Rohatgi, 2020). We collected information from individual studies, where available, on a number of different potential confounding factors in extracted adjusted estimates. This varied by study (see online Supplementary eTable 1) and included current depression diagnosis or symptoms, alcohol use, tobacco use, use of other drugs, educational attainment, psychotic symptoms, socio-economic status, gender, maternal educational level, attention deficit hyperactivity disorder symptoms or diagnosis, maternal substance use during pregnancy, age at initial and follow-up testing, and recency of cannabis use. We extracted final adjusted standardized mean differences that authors reported. Comprehensive information regarding individual study level data is available in the online supplement.

We used the Newcastle-Ottawa Scale to assess the risk of bias in individual studies and present the findings in our results and supplementary materials (Wells et al., 2014). The Newcastle Ottawa Scale is a ten-point rating tool that assesses the quality of selection, comparability and outcome in an individual study. Two authors (EP, AON) calculated the Newcastle Ottawa Scale and agreement was initially 96% (cohen's kappa = 0.9). Following consensus discussion and provision of additional information, the agreement was 100%.

We used the Campbell Collaboration effect size calculator to calculate effect sizes except in linear mixed models where they were calculated in Stata according to Feingold's description (Feingold, 2015; Wilson). We chose *a priori* a random-effects model to estimate the pooled Cohen's *d* statistic. We chose this model due to the expected heterogeneity in study-level characteristics. We calculated the *I*^2^ statistic to measure heterogeneity between studies. We present funnel plots to inspect publication bias and results of the Vevea and Hedges weight-function model for publication bias (Vevea & Hedges, 1995). We used *metan* command function in Stata version 15 for our analysis (Harris et al., 2008).

## Results

We identified 2875 papers and conference abstracts for screening after removal of duplicates. We identified 33 papers for full-text screening. We included seven studies that met our criteria (Fried, Watkinson, & Gray, 2005; Jackson et al., 2016; Meier et al., 2012, 2018; Mokrysz et al., 2016; Ross et al., 2020). (See online Supplementary eFigure 1 for flow chart.)

### Study characteristics

The seven cohorts included in this meta-analysis contain 808 cases and 5308 controls from four Western countries (UK, USA, Canada, New Zealand). We calculated effect sizes from all seven cohorts (see Table 1 for study characteristics). Mean age of follow up was approximately 18 years or less in six/seven studies and at age 38 in one study. Studies varied in their measures of cannabis use; including a mixture of self-report of lifetime total exposure, self-report interval data (past 6 or 12 months) and clinical criteria for past 12-month cannabis-dependency syndrome (see online Supplementary eTable 1). We obtained data from three/seven cohorts from authors for reanalysis (Jackson et al., 2016; Ross et al., 2020). In one case, we could not calculate an effect size from available information. In two further cases, authors presented subtests and multiple categories or cannabis use. We dropped cases from the analysis where subjects' cannabis use was not defined within our preregistered constraints i.e. ever use was recorded without a further specifying amount of use. We pooled data to create one variable including frequent and dependent users where studies utilized more than one measure of cannabis use.

**Table 1.** Study characteristics

Author	Country	Exposed	Controls	Age at follow up (s.d.)	Cannabis effect ‘*d*’ (CI)
Fried et al. (2005)	Canada	38	59	17.86 (1.02)	−0.179 (−0.587 to 0.229)
Jackson et al. (2016)	USA (Minnesota)	308	1387	18.06 (0.63)	−0.136 (−0.234 to −0.038)
Jackson et al. (2016)	USA (RFAB)	118	193	17.89 (0.51)	−0.121 (−0.315 to 0.074)
Mokrysz et al. (2016)	UK	74	1709	15 (not reported)	−0.009 (−0.224 to 0.242)
Meier et al. (2018)	UK	132	1242	18 (not reported)	−0.15 (−0.330 to 0.029)
Meier et al. (2012)	New Zealand	52	242	38 (not reported)	−0.45 (−0.752 to −0.148)
Ross et al. (2020)	USA	86	476	17.25 (0.64)	−0.065 (−0.239–0.108)

### Full-scale IQ decline

We found a significant overall effect for the association between frequent or dependent cannabis use and IQ change [Cohen's *d* = −0.132, (95% CI −0.198 to −0.066) *p* < 0.001]. This corresponds to a 1.98-point decline in IQ (95% CI 0.99–2.97). The *I*^2^ test for heterogeneity was 0.2% indicating low between study statistical heterogeneity. As there were fewer than 10 studies in this analysis, we deemed it inappropriate to conduct Egger's test for publication bias. The Hedges and Vevea weight-function model for publication bias did not indicate any publication bias (see online Supplementary eTable 3). We also generated a funnel plot for this finding (see online Supplementary eFigure 2). Our funnel plot revealed one study marginally outside the pseudo 95% confidence interval; we, therefore, conducted a leave one out sensitivity analysis by rerunning our analysis through multiple iterations sequentially leaving one study out using the *metaninf* command in Stata with random effects. All findings were still statistically significant at *p* < 0.05 indicating that no one study affected the significance of the results overall (see online Supplementary eTable 9). We were unable to disentangle the effects of residual *v.* chronic effects of cannabis as no studies reported this reliably.

### Verbal and performance IQ change

**Fig. 1.**
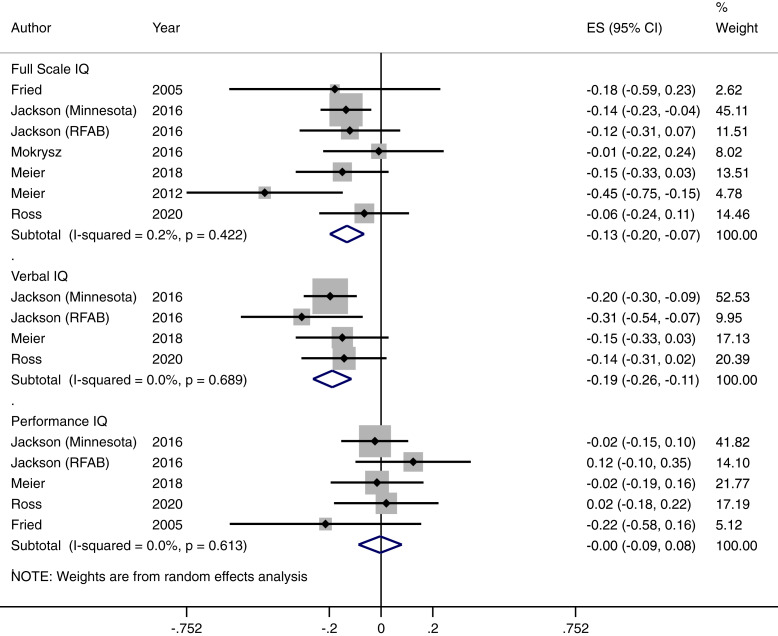
Association between frequent/dependent cannabis use and IQ decline.

In terms of our exploratory analysis, we extracted verbal IQ change effect sizes from four available studies (see online Supplementary eTable 4 for individual study effect sizes). The pooled effect size of verbal IQ decline was *d* = −0.196 CI (−0.27 to −0.122) *p* < 0.001). This was a homogenous finding, *I*^2^ = 0. This corresponds to a decline of 2.94 verbal IQ points CI (1.83–4.05). There was no evidence of performance IQ change following frequent or dependent cannabis use. Estimates of effect sizes were available for five studies (see online Supplementary eTable 5 for individual study effect sizes). The pooled effect size was −0.004 CI = (−0.087–0.080), *p* = 0.938. There was no evidence of heterogeneity in this finding, *I*^2^ = 0. For both verbal and performance IQ change findings, all estimates were within the pseudo 95% confidence intervals within funnel plots, there was no evidence of publication bias from results of the weight-function model and results were not driven by a single study from leave one out sensitivity analysis. See Figure 1, online Supplementary eFigure 6–7; online Supplementary eTable 10–11 for forest plots, funnel plots and leave one out sensitivity analysis tables, respectively.

### Baseline differences in full-scale, verbal and performance IQ

There was no evidence of relative baseline full-scale IQ differences between frequent/dependent cannabis users and non-users (see online Supplementary eTable 6 for individual study estimates & online Supplementary eFigure 7 for Forest plot). The Cohen's *d* statistic was −0.151 CI (−0.325–0.24), *p* = 0.091. This was a heterogeneous finding (*I*^2^ = 78.2%). Individual study effects may have influenced this finding (see online Supplementary eTable 12). Visual inspection of the funnel plot revealed a number of studies outside the pseudo 95% confidence intervals, however, there was no evidence of publication bias from the results of the weight-function model (see online Supplementary eTable 3).

There was no evidence of relative baseline verbal IQ differences between frequent/dependent cannabis users and non-users (see online Supplementary eTable 7 for individual study estimates & online Supplementary eFigure 4 for Forest plot). The Cohen's *d* statistic was −0.164 CI (−0.335–0.008), *p* = 0.061. Study level estimates were available for four studies. There was also significant heterogeneity in this finding (*I*^2^ = 74.2%). Individual study effects may have influenced this finding (see online Supplementary eTable 13). Visual inspection of a funnel plot of this meta-analysis shows study effects outside the pseudo 95% confidence interval (see online Supplementary eFigure 7); however, there was no evidence of publication bias based on findings from the weight-function model (see online Supplementary eTable 3).

There was weak evidence for baseline performance IQ differences in frequent/dependent cannabis users compared to non-users *d* = −0.16, CI (−0.294 to −0.025), *p* = 0.02 (see online Supplementary eTable 8 for individual study estimates & online Supplementary eFigure 5 for Forest plot). Estimates were available for four studies. There was moderate heterogeneity in this finding (*I*^2^ = 57.1%). Visual inspection of the funnel plot revealed one study outside the pseudo 95% confidence interval (see online Supplementary eFigure 8), however, there was no evidence of publication bias from findings of the weight-function model (see online Supplementary eTable 3). Individual study effects may have influenced this finding (see online Supplementary eTable 14 for leave one out sensitivity analysis).

### Quality assessment

We found that individual studies had adequate to excellent quality. Two studies scored 10/10 and 9/10 each on quality indices, a further three studies scored 8/10 and two studies had an adequate quality of 6/10. Overall, 93% of quality indices were met in definition and selection of case and control subjects, 43% of quality indices were met in comparability of case and control subjects and 82% of quality indices were met for assessment of outcome across studies. Measurement of cannabis use varied by study, however, most studies used semi-structured tools and only two studies relied completely on self-report questionnaires. Evidence also supports adjusting for recent use of cannabis, as well as problem alcohol use and other drug use, and these were the covariates we selected for scoring the comparability arm of the quality assessment. One study provided an estimate accounting for recent use of cannabis; however, they were unable to provide an accurate estimate of this effect due to model selection issues and heteroskedastic data (P. A. Fried et al., 2005). Five/seven studies accounted for problem use of alcohol or other drug use. Three/seven studies adjusted for tobacco use. All studies adjusted for sex. We deemed retention of >70% to be acceptable and this was achieved by five/seven studies (see online Supplementary eTable 1 and 2 for quality assessment and further description of adjustments in extracted estimates). Agreement between raters was on 96.43% of items with a Cohen's kappa value of 0.9 indicating excellent agreement. Disagreements were resolved with additional information and consensus discussion. Overall, the quality assessment revealed that lack of classification of residual and chronic effects of cannabis use separately was persistent in the longitudinal literature.

## Discussion

This is the first longitudinal quantitative synthesis to our knowledge examining the association between frequent or dependent cannabis use during adolescence and IQ change over time. We found that young people who use cannabis frequently or dependently by age 18 have declined in IQ at follow up and this may be due to a decline in verbal IQ. All studies showed point estimates of IQ decline. Our inclusion criteria were broad and the cannabis-using cohort represents a spectrum of intensity of use. Findings from our exploratory analysis indicate that there were no differences between pre-cannabis exposure IQ of cannabis users compared to control subjects, however, this was a heterogeneous finding.

We note the likely duration of exposure prior to follow up in this study are relatively short in six of seven cohorts given that the age of follow up was limited to adolescence in these studies. The approximately 2-point decline in IQ in adolescent-onset frequent cannabis users is not to be clinically significant and alone is unlikely to completely explain a range of psychosocial problems linked to cannabis use in this cohort. Developmental effects, however, such as altered neuromaturational processes may not be fully captured by periods of follow up limited to adolescence when brain development is ongoing (Westlye et al., 2009). There is sparse data examining persistent heavy cannabis use from youth over longer periods, and no longitudinal data examining IQ from cannabis use onset in youth between 18 and 25 years. Specifically, this finding is not likely to be attributable to alcohol use. Most studies in this meta-analysis controlled for alcohol use. Previous research also shows that a network of both predisposing, co-occurring and lagged cognitive effects are associated with cannabis use and are also likely to have additive effects above that of alcohol (Morin et al., 2019). Educational engagement may be an influencing factor and represents one potential pathway to IQ decline (Castellanos-Ryan et al., 2017). Quasi-experimental evidence has found associations between cannabis availability and educational performance in college-age young people and this may represent a potential mechanism (Marie & Zölitz, 2017). Other social vulnerability factors such as pre-exposure reading ability and years of education, however, may also explain the effects found (Brinch & Galloway, 2012; Price, Ramsden, Hope, Friston, & Seghier, 2013).

Our findings are characteristically similar to the premorbid loss of intelligence in schizophrenia, a disorder with a known neurodevelopmental aetiology. Verbal IQ declines, by approximately 6 points have been found in males who are later diagnosed with schizophrenia between ages 13 and 17 premorbid, with no relative decline found in performance IQ (MacCabe et al., 2013). The effect seen in this study is similar in size to effect sizes seen in exposure to lead, an environmental toxin, in childhood (Reuben et al., 2017).

### Other considerations

There is limited data on the effects of cannabis on higher-order cognitive processing, i.e. executive functioning. The relationship between IQ and executive functioning is complex and further research examining executive functioning development and cannabis is important, particularly given the role of inhibitory control in the aetiology of substance use disorders in general (Friedman et al., 2006; Ross et al., 2020). Effects on executive functioning such as inhibitory control may be more markedly affected by cannabis use in adolescence and these within-person effects are sustained beyond a 12-month abstinence period (Castellanos-Ryan et al., 2017; Morin et al., 2019). Fried and colleagues previously found that offspring of women who smoke cannabis during pregnancy exhibit executive functioning deficits later in childhood (Fried & Smith, 2001). Cannabinoid 1 (CB1) receptors (the main receptors responsible for mediating the effects of cannabis in the brain) are concentrated primarily in the hippocampus, frontal cortex and cerebellum – areas important to executive function development. CB1 receptors are more densely expressed in earlier life than in adulthood in these regions where they are known to play a role in synaptic pruning during development (Lubman et al., 2015; Orr, Paschall, & Banich, 2016). Studies have also found that cannabis is associated with loss of white matter integrity (Orr et al., 2016). Triangulating this evidence, animal model data also supports this hypothesis (Rubino et al., 2015). Future studies would benefit from including diverse measures of cognition in research on the neurodevelopmental effects of cannabis use in adolescence, specifically as they can be informative about mechanisms for substance use disorders more widely.

### Covariate selection

Decisions on covariate selection varied widely by the research group. Meier and colleagues adjusted for many covariates; however, we were only able to extract an estimate adjusted for sex. In their 2012 paper, they reported that persisting dependence from adolescence to midlife was associated with a clinically relevant decline in IQ, a total of 8 points in multiwave cannabis-dependent middle-age adults who had an initial past 12-month diagnosis of cannabis dependence at 18 (Meier et al., 2012, 2018). To our knowledge, this study has not been replicated since. The excellent retention profile of this cohort study also raises concerns about the effects of the degree to which attrition bias may influence the magnitude of the findings in other studies. The use of tobacco as a covariate is problematic, as it is almost universally used with cannabis, making the disentangling of causal effects difficult. Use of penalized regression models or propensity score matching in future studies may help overcome some of these issues. Mendelian randomization also offers another avenue to investigate potential causal associations between frequent and dependent cannabis use and IQ, and is an approach that can potentially disentangle the effects of tobacco use.

### Measurement issues

Reliance on self-report data in substance use research in general is a significant source of measurement error. Social desirability biases, panel conditioning effects and high rates of recanting in previous studies also compromise the accuracy of non-corroborated self-report data (Percy, McAlister, Higgins, McCrystal, & Thornton, 2005). This may effect inference of accurate dose–response relationships. Whilst robust quantitative biological methods to detect drug use have not been developed, hair and urine analyses offer promise to corroborate self-reports particularly in quantifying recent use (urine) and heavy use (hair) (Donovan et al., 2012; Taylor et al., 2017). Repeated measurement of substance use may also improve sensitivity. Recall bias is also demonstratively important: some studies opted to ask individuals for lifetime estimates of drug use and others asked about past 6- or 12-month frequency of use. Repeated measures over 6 or 12 monthly periods may be of benefit in future studies. (Donovan et al., 2012)

### Strengths

This study has many key strengths. The use of comparable outcome measures by constituent studies is relevant to the robustness of the findings. Our stringent case inclusion criteria intended to capture individuals with reliably moderate to high levels of use, diminishing the effects of capturing potential false-positive cases. Our longitudinal design is novel and has not been previously undertaken.

### Limitations

There are two differences between our preregistration and our study. Firstly, we did not examine specific cognitive functions as outlined in our preregistration due to data accessibility reasons but assessed full-scale IQ, and verbal and performance IQ. Secondly, we were unable to exclude studies that did not account for the recent use of cannabis. This is a potential confounding factor and results seen in this study may be due to residual rather than chronic effects of cannabis. Only one study measured this appropriately, however, the authors were unable to provide an accurate estimate from this data (Fried et al., 2005). Residual effects may last for up to 28 days and the existence of chronic effects would have significant public health implications. Adjusting for recent cannabis use in non-randomized cohort data, however, may not address this problem. Subjects who have the heaviest use patterns and are potentially most cognitively impaired would be least likely to abstain from cannabis for significant periods prior to testing. The finding that there is no longitudinal data investigating post-residual chronic effects of cannabis on IQ in youth is concerning given international drug policy changes. The IQ decline whilst modest in size is in the context of ongoing neurodevelopment. As most studies had limited periods of follow up and given the chronicity of cannabis use, our study may underestimate overall potential developmental effects. Finally, studies did not provide reliable change index estimates and this would improve the validity of the findings, i.e. the findings of change in IQ are not due to measurement error. This should be an important consideration in improving the quality of future research.

In summary, this systematic review and meta-analysis show evidence for an almost 2-point decline in IQ associated with frequent or dependent cannabis use in adolescence. The majority of studies included (six/seven) had follow up in the mid to late teens while brain development is still occurring thus limiting the interpretation of developmental impact. One study which had follow up in mid-life had a greater magnitude finding with a dose–response relationship, indicating that a potential neurodevelopmental impact of cannabis use in adolescence may be underestimated by our systematic review as follow-up periods in the current literature are limited. Our findings could be explained by several potential mechanisms: a developmental neurotoxicity mechanism, a social pathway influenced by deviancy and educational non-engagement, by residual effects of cannabis or by individual vulnerability factors such as reading ability in childhood or by genetic factors. Hypotheses such as family level vulnerability predisposing to IQ decline are possible but are less likely to be fully explanatory (Ellingson et al., 2020). Adolescence and early adulthood are crucial periods for completing education and establishing career trajectories and social relationships for later in life and given the negative effects of cannabis use in this age group, reducing the prevalence of its use should remain a priority (Patel, Flisher, Hetrick, & McGorry, 2007).

## Supplementary material

For supplementary material accompanying this paper visit https://doi.org/10.1017/S0033291720005036. click here to view supplementary material
