# Supplementary material for: Intelligence quotient decline following frequent or dependent cannabis use in youth: a systematic review and meta-analysis of longitudinal studies
Source: Psychol Med. 2021 Jan 27;51(2):194–200. doi: 10.1017/S0033291720005036 (PMC7893511; doi:10.1017/S0033291720005036)
Supplement: Supplementary file 1 [file S0033291720005036sup001.docx]

**Supplementary Materials:**

**Search Strategy**

Search strategy for PubMed:

A and B and C ((("Cognitive Dysfunction"[Mesh] OR "Cognition"[Mesh] OR "Executive Function"[Mesh] OR "Cognitive Reserve"[Mesh] OR "Cognitive Neuroscience"[Mesh] OR "Neurocognitive Disorders"[Mesh] OR neurocognitive[Title/Abstract] OR cognition[Title/Abstract] OR cognitive[Title/Abstract] OR "Neuropsychology"[Mesh] OR "Neuropsychological Tests"[Mesh] OR "Intelligence"[Mesh] OR "Memory, Short-Term"[Mesh] OR "Memory, Long-Term"[Mesh] OR "Memory"[Mesh] OR "Mental Recall"[Mesh] OR mental[Title/Abstract] OR "Mental Processes"[Mesh] OR neuropsychology[Title/Abstract] OR attention[title/abstract] OR memory[title/abstract] OR intelligence[title/abstract])) AND (("Cannabis"[Mesh] OR cannabis[Title/Abstract] OR "Marijuana Smoking"[Mesh] OR "Marijuana Abuse"[Mesh] OR marijuana[Title/Abstract] OR tetra-hydrocannabinol[Title/Abstract]))) AND (("cohort studies"[MeSH Terms:noexp] OR "longitudinal studies"[MeSH Terms:noexp] OR "follow-up studies"[MeSH Terms:noexp] OR "prospective studies"[MeSH Terms:noexp] OR "retrospective studies"[MeSH Terms:noexp] OR "Comparative Study" [Publication Type] OR cohort[TIAB] OR longitudinal[TIAB] OR prospective[TIAB] OR retrospective[TIAB] OR comparative study[TIAB])) 1932

C "cohort studies"[MeSH Terms:noexp] OR "longitudinal studies"[MeSH Terms:noexp] OR "follow-up studies"[MeSH Terms:noexp] OR "prospective studies"[MeSH Terms:noexp] OR "retrospective studies"[MeSH Terms:noexp] OR "Comparative Study" [Publication Type] OR cohort[TIAB] OR longitudinal[TIAB] OR prospective[TIAB] OR retrospective[TIAB] OR comparative study[TIAB] 3890196

B "Cannabis"[Mesh] OR cannabis[Title/Abstract] OR "Marijuana Smoking"[Mesh] OR "Marijuana Abuse"[Mesh] OR marijuana[Title/Abstract] OR tetra-hydrocannabinol[Title/Abstract] 29236

A "Cognitive Dysfunction"[Mesh] OR "Cognition"[Mesh] OR "Executive Function"[Mesh] OR "Cognitive Reserve"[Mesh] OR "Cognitive Neuroscience"[Mesh] OR "Neurocognitive Disorders"[Mesh] OR neurocognitive[Title/Abstract] OR cognition[Title/Abstract] OR cognitive[Title/Abstract] OR "Neuropsychology"[Mesh] OR "Neuropsychological Tests"[Mesh] OR "Intelligence"[Mesh] OR "Memory, Short-Term"[Mesh] OR "Memory, Long-Term"[Mesh] OR "Memory"[Mesh] OR "Mental Recall"[Mesh] OR mental[Title/Abstract] OR "Mental Processes"[Mesh] OR neuropsychology[Title/Abstract] OR attention[title/abstract] OR memory[title/abstract] OR intelligence[title/abstract]

Search strategy for Embase

A1: 'cognition'/exp OR 'cognitive function test'/exp OR 'cognition assessment'/exp OR 'executive function'/exp OR 'metacognition'/exp OR 'mental capacity'/exp OR 'mental performance'/exp OR 'neuropsychology'/exp OR 'memory'/exp OR 'intelligence'/exp OR 'attention'/exp

A2: cognition:ti,ab OR cognitive:ti,ab OR metacognition:ti,ab OR mental:ti,ab OR neuropsychology:ti,ab OR memory:ti,ab OR intelligence:ti,ab OR attention:ti,ab

B: A1 OR A2

C: 'cannabis'/exp OR 'cannabis addiction'/exp OR 'cannabis use'/exp OR cannabis:ti,ab OR marijuana:ti,ab OR 'tetra hydrocannabinol':ti,ab

D: 'cohort analysis'/exp OR cohort:ti,ab OR 'retrospective study'/exp OR retrospective:ti,ab OR 'longitudinal study'/exp OR longitudinal OR 'prospective study'/exp OR prospective:ti,ab OR 'follow up'/exp OR 'comparative study'/exp OR comparative:ti,ab

Combine results: B AND C AND D

Search strategy for PsychInfo

A1: DE "Cognition" OR DE "Cognitive Impairment" OR DE "Cognitive Assessment" OR DE "Cognitive Reserve" OR DE "Cognitive Processes" OR DE "Cognitive Development"DE "Memory" OR DE "Short Term Memory" OR DE "Memory Decay" OR DE "Memory Disorders" OR DE "Neurocognitive Disorders" OR DE "Neurocognition"DE "Attention" OR DE "Attention Span" OR DE "Intelligence" OR DE "Neuropsychological Assessment"

A2: ( TI Cognition OR TI Cognitive OR Memory OR TI Neurocognitive OR TI Attention TI Intelligence OR TI neuropsychological ) OR ( AB Cognition OR AB Cognitive OR AB Memory OR AB Neurocognitive OR AB Attention AB Intelligence OR AB neuropsychological )

B: A1 OR A2

C: DE "Cannabis" OR DE "Cannabis Use Disorder" OR TI cannabis OR AB cannabis OR DE "Marijuana" OR DE "Marijuana Usage" OR TI marijuana OR AB marijuana OR tetrahydrocannabinol

D: DE "Cohort Analysis" OR TI cohort OR AB cohort OR DE "Prospective Studies" OR TI prospective OR AB prospective OR OR DE "Retrospective Studies" OR TI Retrospective OR AB retrospectibe OR DE "Longitudinal Studies" OR TI Longitudina OR AB Longitudina OR TI comparative OR AB comparative OR TI followup OR AB followup

Combine Results: B AND C AND D

eTable 1: Study characteristics

| Study | 1 | 2 | 3 | 4 | 5 | 6 | 7 |
| --- | --- | --- | --- | --- | --- | --- | --- |
| Author | Fried | Jackson (MTFS) | Jackson (RFAB) | Mokryz | Meier | Meier | Ross |
| Year | 2005 | 2016 | 2016 | 2016 | 2018 | 2012 | 2020 |
| Baseline IQ | WISC-III (Wechsler, 1991) | Vocabulary, Information, Block Design, and Picture Arrangement from the WISC-R (Wechsler, 1974) | Vocabulary, Similarites, Block Design, and Matrix Reasoning from the WISC-R (Wechsler, 1974) | WISC-III  (Wechsler, 1991) | WISC-R  (Wechsler, 1974) | WISC-R (Wechsler, 1974) | Average of 3 tests: At 4 years Stanford-Binet (Terman & Merrill, 1973), at 7 years WISC-R (Wechsler, 1974), and 12 years WISC-III (Wechsler, 1991) |
| Follow up IQ | WAIS-III  (Wechsler, 1997) | Vocabulary, Information, Block Design, and Picture Arrangement from WAIS-R (Wechsler D, 2008) | Vocabulary, Similatirites, Block Design, and Matrix Reasoning from WAIS-R (Wechsler D, 2008) | WASI (Matrix and Vocabulary subtest composite) (Weschsler D, 1991) | WAIS-IV  (Weschler D, 2008) | WAIS-IV  (Weschler D, 2008) | WAIS-III  (Wechsler, 1997) |
| Baseline Age | 9-12 | 11-12 | 9-10 | 8 | 12 | 7, 9, 11, 13 | 4, 7, 12 |
| Follow up age | 17-21 | 17-20 | 17-20 | 15 | 18 | 38 | 16-20 |
| Cannabis exposure | 3679 joints (lifetime measure – mean exposure) | >30 uses lifetime (61% of these used cannabis daily for at least 6 months) | >30 uses lifetime  (of whom 40% used cannabis daily for at least 6 months) | ≥50 lifetime uses | At least weekly use | Multiwave measures of past 12 month dependence | Data fit to preregistration compositing variables on monthly use, estimated number of days in the past 180 days and DSM criteria for cannabis use disorder. Of note low number endorsing daily use of cannabis (n=16). |
| Substance use measure | Urine drug testing and self report | CIDI-SAM semi-structured interview | Self-report questionnaire | Self-report questionnaire | Semi-structured interview | Semi-structured interview | CIDI-SAM semi-structured interview |
| Control Exposure | No cannabis use | No cannabis exposure | No cannabis exposure | No cannabis exposure | No cannabis exposure | No cannabis exposure | No cannabis exposure |
| Study type | Prospective birth cohort | Twin study prospective cohort | Twin study prospective cohort | Prospective birth cohort | Co-twin control prospective cohort | Prospective birth cohort | Co-twin control prospective cohort |
| Location | Canada | USA | USA | UK | UK | New Zealand | USA |
| Accounts for recent use | Yes | No | No | No (reports 47.3% of case sample have used in the past 3 days) | No | No | No |
| Cases | 38 | 308 | 118 | 74 | 132 | 52 | 86 |
| Control | 59 | 1387 | 193 | 1709 | 1242 | 242 | 476 |
| Year | 2005 | 2016 | 2016 | 2015 | 2018 | 2012 | 2020 |
| Retention | 75% follow up | 73% had complete available data for inclusion | 58% had complete available data for inclusion | 16% of original cohort with complete data for inclusion. Authors used multiple imputation using Stata ice command assuming missingness at random in additional analysis | 96% follow up | 89% follow up | 84% follow up |
| **Covariates included in adjusted estimates** | | | | | | | |
| Alcohol | Number of drinks week of study | Adjusted estimate includes control for self-reported history of binge drinking coded as dichotomous | Adjusted estimate includes control for self-reported history of binge drinking coded as dichotomous | Adjusted estimate controls for ordinal scale of self reported interval lifetime uses “never, 1–5, 6–19, 20–39, 40–99, ⩾100 times” | Not adjusted for in estimate. | Post-hoc sensitivity analysis, not included in analysis, not adjusted for in estimate. | Adjusted estimate includes controls for alcohol dependency |
| Cigarettes | Included as covariate (current cigs per day) | Not accounted for | Not accounted for | Adjusted estimate controls for ordinal scale of self reported interval lifetime uses “never, 1–5, 6–19, 20–39, 40–99, ⩾100 times” | Not accounted for | Not accounted for | Included as covariate – dichotomous variable – regular tobacco use |
| Other drugs | Excluded from analysis | Included as covariate (dichotomous variable – lifetime use) | Included as covariate (dichotomous variable – lifetime use) | Included as ordinal covariate “one drug/ more than one drug/ none” | Not adjusted for in estimate | Post-hoc sensitivity analysis, not included in analysis, not adjusted for in estimate. | Included as covariate – other drug dependence, dichotomous variable |
| Psychopathology | Any DSM axis 1 diagnosis measured by computer assessment tool. (collapsed into dichotomous variable) | Not accounted for in analysis | Not accounted for in analysis | Adjusted separately for parent reported hyperactivity and conduct subscales of SDQ at 11 years, Short Moods and Feelings Questionnaire at 12 years and psychotic symptoms (PLIKSi interview) at 12 years. | Not adjusted for in estimate | Post-hoc sensitivity analysis, not included in analysis, not adjusted for in estimate. | Not adjusted for in estimate |
| SES | Parental educational level, family income | Hollingshead index/ parental education/ household income composite measure | Hollingshead index | Maternal educational level | Not adjusted for in estimate | Post-hoc sensitivity analysis, not included in analysis, not adjusted for in estimate. | Not adjusted for in fixed effects estimate |
| Other covariates included in analysis | Maternal use of alcohol, cigarettes and cannabis during pregnancy | n/a | n/a | Maternal depressive symptoms during pregnancy and up to 8 months post natal using Crown-Crisp experiential index, maternal alcohol (ordinal)/ cigarette (dichotomous) and cannabis (dichotomous) use in pregnancy. Maternal suspected truancy at 14 years. (dichotomous) | n/a | n/a | n/a |

eTable 2: Newcastle Ottawa scale

| Quality Indices | | Study ID Number | | | | | | | Total Score | Domain Score |
| --- | --- | --- | --- | --- | --- | --- | --- | --- | --- | --- |
|  |  | Fried (2005) | Jackson  (2016 -MTFS) | Jackson  (2016 - RFAB) | Mokryz  (2016) | Meier (2018) | Meier (2012) | Ross  (2020) |  |  |
| Selection | Representativeness of exposed cohort | ★ | ★ | ★ | ★ | ★ | ★ | ★ | 7/7 | 93% |
|  | Selection of non-exposed cohort | ★ | ★ | ★ | ★ | ★ | ★ | ★ | 7/7 |  |
|  | Ascertainment of exposure (Star for semi-structured interview) | ★ | ★ |  |  | ★ | ★ | ★ | 5/7 |  |
|  | Outcome not present at start | ★ | ★ | ★ | ★ | ★ | ★ | ★ | 7/7 |  |
| Comparability | Adequate controls  (adjusts for recent use - one star alcohol/other substances – one star) | ★★ | ★ | ★ | ★ |  |  | ★ | 6/14 | 43% |
| Outcome | Assessment of outcome  (FSIQ: two stars, composite of verbal and non-verbal subtest one star, other composite measure zero stars) | ★★ | ★ | ★ | ★ | ★★ | ★★ | ★★ | 11/14 | 82% |
|  | Adequate Follow up period (>2 yrs) | ★ | ★ | ★ | ★ | ★ | ★ | ★ | 7/7 |  |
|  | Study retention (>70%) | ★ | ★ |  |  | ★ | ★ | ★ | 5/7 |  |
| Total Score | | 10/10 | 8/10 | 6/10 | 6/10 | 8/10 | 8/10 | 9/10 | 55/70 | 79% |

eTable 3: Results from The Vevea and Hedges Weight-Function Model for Publication Bias

| Meta-analysis | Unadjusted likelihood | Adjusted likelihood | Difference | p value |
| --- | --- | --- | --- | --- |
| FSIQ Change | -12.2 | -12.2 | 0.019 | 0.889 |
| VIQ Change | -9.27 | -9.27 | 0 | 0.985 |
| PIQ Change | -7.53 | -7.63 | 0.209 | 0.647 |
| FSIQ Baseline | -5.58 | -5.92 | 0.68 | 0.41 |
| VIQ Baseline | -5.42 | -5.49 | 0.15 | 0.699 |
| PIQ Baseline | -5.43 | -5.48 | 0.106 | 0.744 |

eTable 4: Individual study effects of associations between frequent/dependent cannabis use and VIQ change

| Author | Country | Exposed | Controls | Age at follow up (SD) | Cannabis Effect *“d”* (CI) |
| --- | --- | --- | --- | --- | --- |
| Jackson (2016) | USA (Minnesota) | 308 | 1387 | 18.06 (0.63) | -0.197 (-0.299- -0.094) |
| Jackson (2016) | USA (RFAB) | 118 | 193 | 17.89 (0.51) | -0.307 (-0.542- -0.071) |
| Meier (2018) | UK | 132 | 1242 | 18 (not reported) | -0.15 (-0.330- 0.029) |
| Ross (2020) | USA | 86 | 476 | 17.25 (0.64) | -0.143 (-0.308- 0.021) |

eTable 5: Individual study effects of associations between frequent/dependent cannabis use PIQ change

| Author | Country | Exposed | Controls | Age at follow up (SD) | Cannabis Effect *“d”* (CI) |
| --- | --- | --- | --- | --- | --- |
| Jackson (2016) | USA (Minnesota) | 308 | 1387 | 18.06 (0.63) | -0.024 (-0.154 - 0.105) |
| Jackson (2016) | USA (RFAB) | 118 | 193 | 17.89 (0.51) | 0.124 (-0.099 - 0.347) |
| Meier (2018) | UK | 132 | 1242 | 18 (not reported) | -0.016 ( -0.195 - 0.164) |
| Ross (2020) | USA | 86 | 476 | 17.25 (0.64) | 0.020 (-0.182 - 0.222) |
| Fried (2005) | Canada | 38 | 59 | 17.86 (1.02) | -0.215 (-0.585 - 0.155) |

eTable 6: Individual study effects of associations between frequent/dependent cannabis use and FSIQ baseline

| Author | Country | Exposed | Controls | Age at follow up (SD) | Cannabis Effect *“d”* (CI) |
| --- | --- | --- | --- | --- | --- |
| Jackson (2016) | USA (Minnesota) | 308 | 1387 | 18.06 (0.63) | -0.106 (-0.229 - 0.018) |
| Jackson (2016) | USA (RFAB) | 118 | 193 | 17.89 (0.51) | -0.024 (-0.204 - 0.154) |
| Meier (2018) | UK | 132 | 1242 | 18 (not reported) | -0.373 (-0.553- -0.193) |
| Ross (2020) | USA | 86 | 476 | 17.25 (0.64) | -0.388 (-0.618 - -0.157) |
| Mokrysz (2016) | UK | 74 | 1709 | 15 (not reported) | 0.140 (-0.093 - 0.373) |

eTable 7: Individual study effects of associations between frequent/dependent cannabis use and VIQ baseline

| Author | Country | Exposed | Controls | Age at follow up (SD) | Cannabis Effect *“d”* (CI) |
| --- | --- | --- | --- | --- | --- |
| Jackson (2016) | USA (Minnesota) | 308 | 1387 | 18.06 (0.63) | -0.147 ( -0.271 - -0.024) |
| Jackson (2016) | USA (RFAB) | 118 | 193 | 17.89 (0.51) | 0.043 (-0.136 - 0.222) |
| Meier (2018) | UK | 132 | 1242 | 18 (not reported) | -0.397 ( -0.577 - -0.217) |
| Ross (2020) | USA | 86 | 476 | 17.25 (0.64) | -0.155 ( -0.390 - 0.079) |

eTable 8: Individual study effects of associations between frequent/dependent cannabis use and PIQ Baseline

| Author | Country | Exposed | Controls | Age at follow up (SD) | Effect size (CI) |
| --- | --- | --- | --- | --- | --- |
| Jackson (2016) | USA (Minnesota) | 308 | 1387 | 18.06 (0.63) | -0.024 ( -0.147- 0.099) |
| Jackson (2016) | USA (RFAB) | 118 | 193 | 17.89 (0.51) | -0.072 (-0.252- 0.108) |
| Meier (2018) | UK | 132 | 1242 | 18 (not reported) | -0.204 -0.384 -0.025 |
| Ross (2020) | USA | 86 | 476 | 17.25 (0.64) | -0.405 (-0.641- -0.170) |
| Fried (2005) | Canada | 38 | 59 | 17.86 (1.02) | -0.215 (-0.585- 0.155) |

eTable 9: Leave one out sensitivity analysis for association between frequent/dependent cannabis use and FSIQ change

| Study left out | Effect Size (d) | CI |
| --- | --- | --- |
| Jackson (Minnesota) | -.134 | -.233- -.035 |
| Jackson (RFAB) | -.136 | -.219- -.054 |
| Ross | -.145 | -.22- -.071 |
| Fried | -.132 | -.209- -.056 |
| Meier (2018) | -.131 | -.215- -.048 |
| Meier (2012) | -.117 | -.185- -.049 |
| Mokrysz | -.142 | -.211- -.074 |

eTable 10: Leave one out sensitivity analysis for association between frequent/dependent cannabis use and VIQ change

| Study left out | Effect Size (d) | CI |
| --- | --- | --- |
| Jackson (Minnesota) | -.195 | -.303- -.087 |
| Jackson (RFAB) | -.183 | -.262- -.105 |
| Ross | -.21 | -.293- -.126 |
| Meier (2018) | -.197 | -.279- -.115 |

eTable 11: Leave one out sensitivity analysis for association between frequent/dependent cannabis use and PIQ change

| Study left out | Effect Size (d) | CI |
| --- | --- | --- |
| Jackson (Minnesota) | .011 | -.099- .121 |
| Jackson (RFAB) | -.024 | -.115- .066 |
| Ross | -.008 | -.1- .083 |
| Fried | .008 | -.078- .094 |
| Meier (2018) | 0 | -.095- .094 |

eTable 12: Leave one out sensitivity analysis for association between frequent/dependent cannabis use and FSIQ baseline

| Study left out | Effect Size | CI |
| --- | --- | --- |
| Jackson (Minnesota) | -.163 | -.41- .084 |
| Jackson (RFAB) | -.183 | -.398- .032 |
| Ross | -.099 | -.284- .085 |
| Meier (2018) | 0 | -.095- .094 |
| Mokrysz | -.212 | -.384- -.04 |

eTable 13: Leave one out sensitivity analysis for association between frequent/dependent cannabis use and VIQ baseline

| Study left out | Effect Size | CI |
| --- | --- | --- |
| Jackson (Minnesota) | -.17 | -.443- .102 |
| Jackson (RFAB) | -.231 | -.397- -.066 |
| Ross | -.166 | -.389- .056 |
| Meier (2018) | -.089 | -.215- .036 |

eTable 14: Leave one out sensitivity analysis for association between frequent/dependent cannabis use and PIQ baseline

| Study left out | Effect Size | CI |
| --- | --- | --- |
| Jackson (Minnesota) | -.210 | -.353- -.068 |
| Jackson (RFAB) | -.192 | -.37- -.015 |
| Ross | -.088 | -.177- .001 |
| Fried | -.156 | -.308- -.005 |
| Meier (2018) | -.153 | -.325- .0183 |

eFigure 1: Prisma flow diagram

R

ecords identified through

database searching

(

n =

5815

)

Records

after duplicates removed

n =

(

2875

)

Records

screened

(

n =

2875

)

R

ecords excluded

(

n =

2841

)

F

ull

-

text articles assessed

for eligibility

n =

(

33

)

F

ull

-

text articles excluded, with

reasons

Cognitive measure

not IQ

N=9

Substance use at baseline N=6

Preliminary Report N=4

Synthetic Cannabinoids N=2

C

ross

-

sectional study N=2

Perspective article N=1

No response from author N=1

Cannabis measured as lifetime N=1

S

tudies included in

qualitative synthesis

n =

(

7

)

S

tudies included in

quantitative synthesis

(

meta

-

analysis)

(

n =

7

)

0

.05

.1

.15

.2

v FSIQ change

-.6

-.4

-.2

0

.2

d FSIQ change

Figure 2: FSIQ change funnel plot with pseudo 95% confidence limits


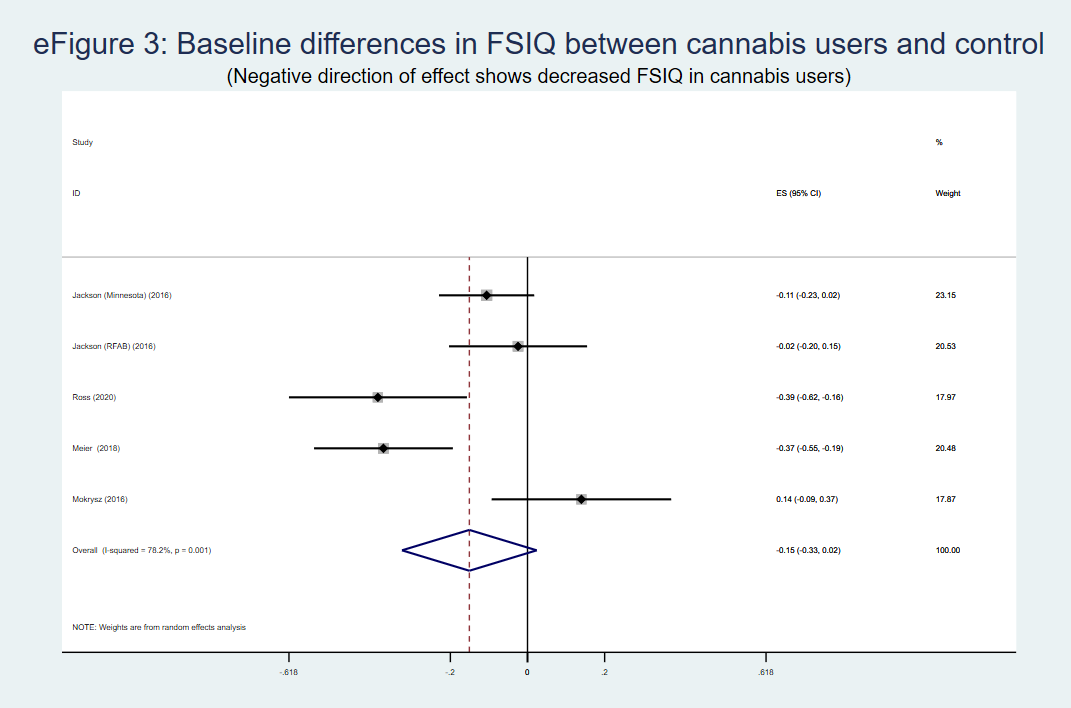

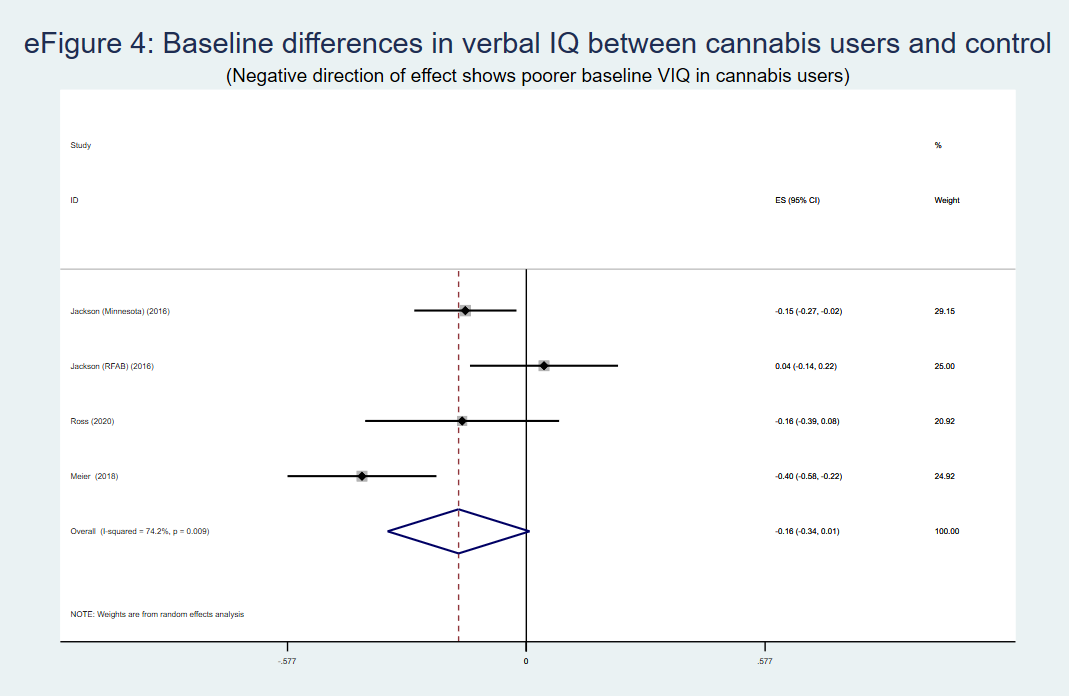

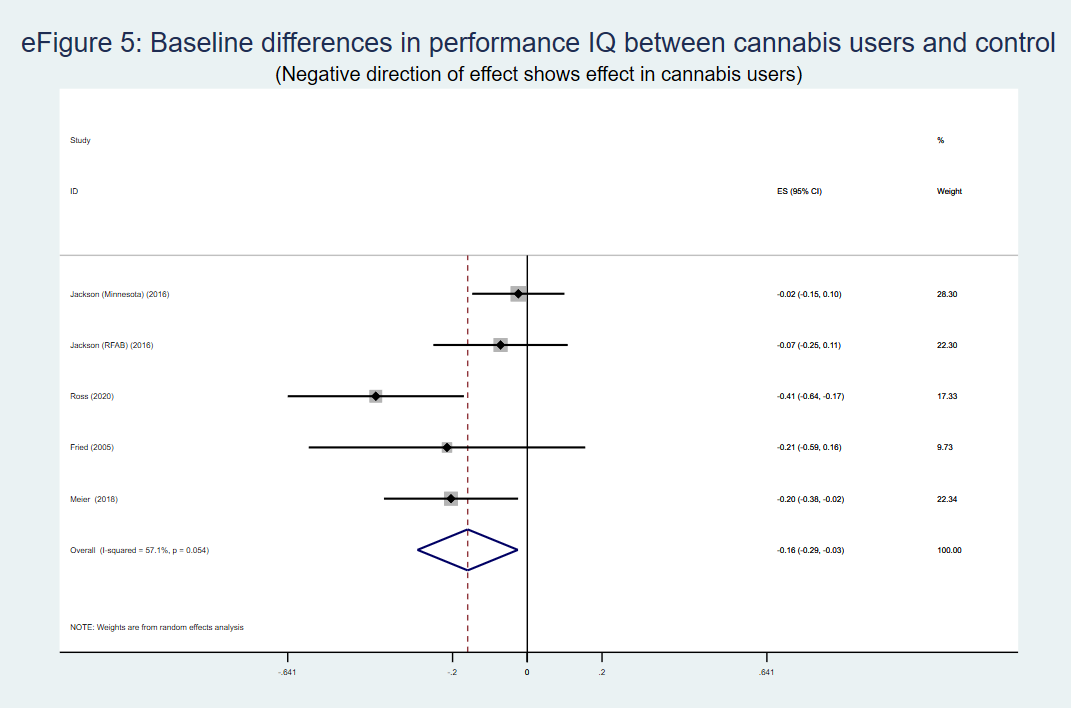


eFigure 6:


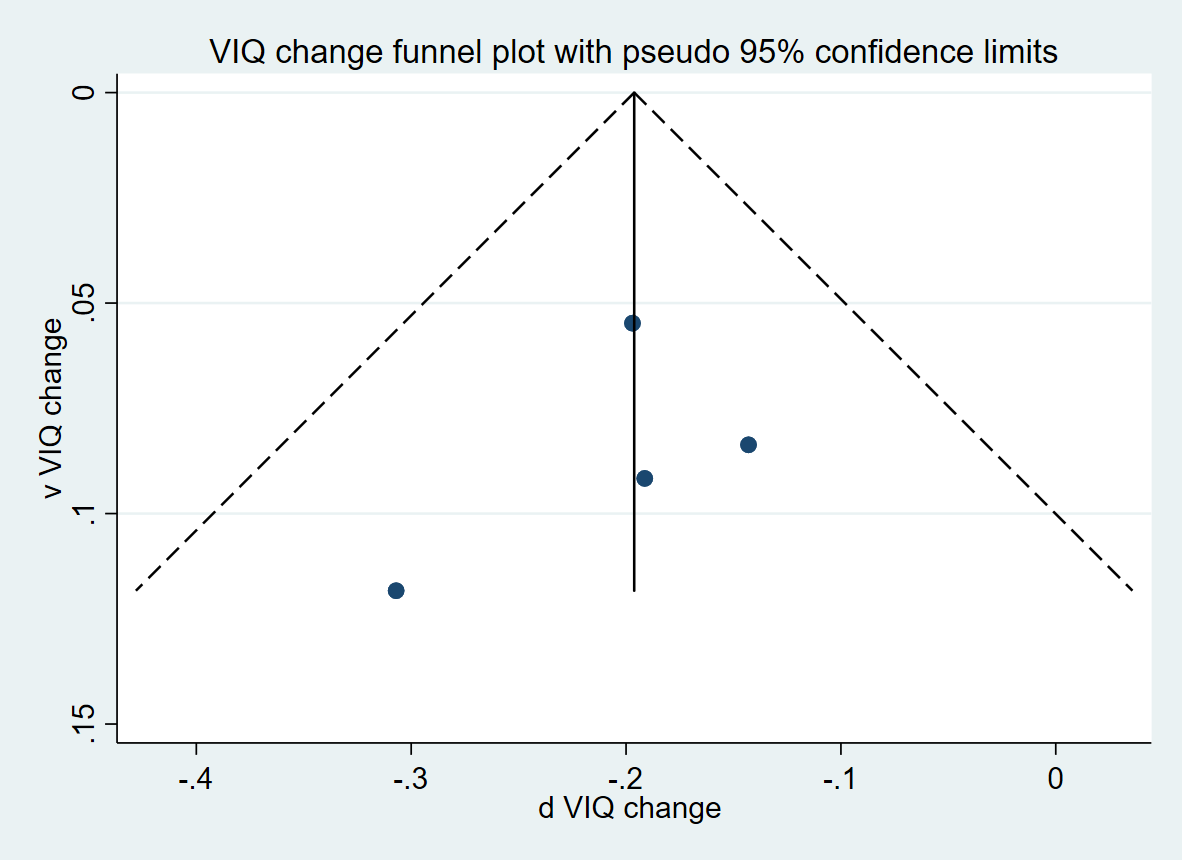


eFigure 7:


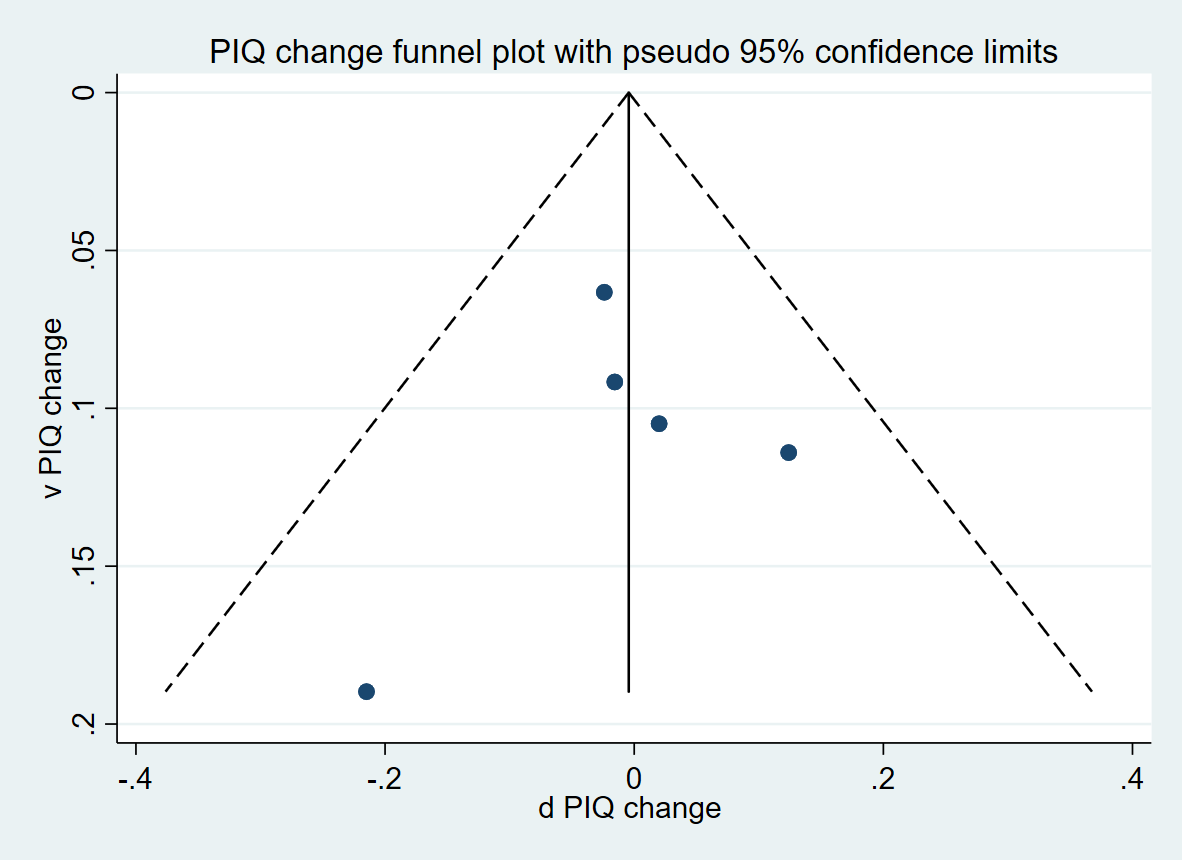


eFigure 8:


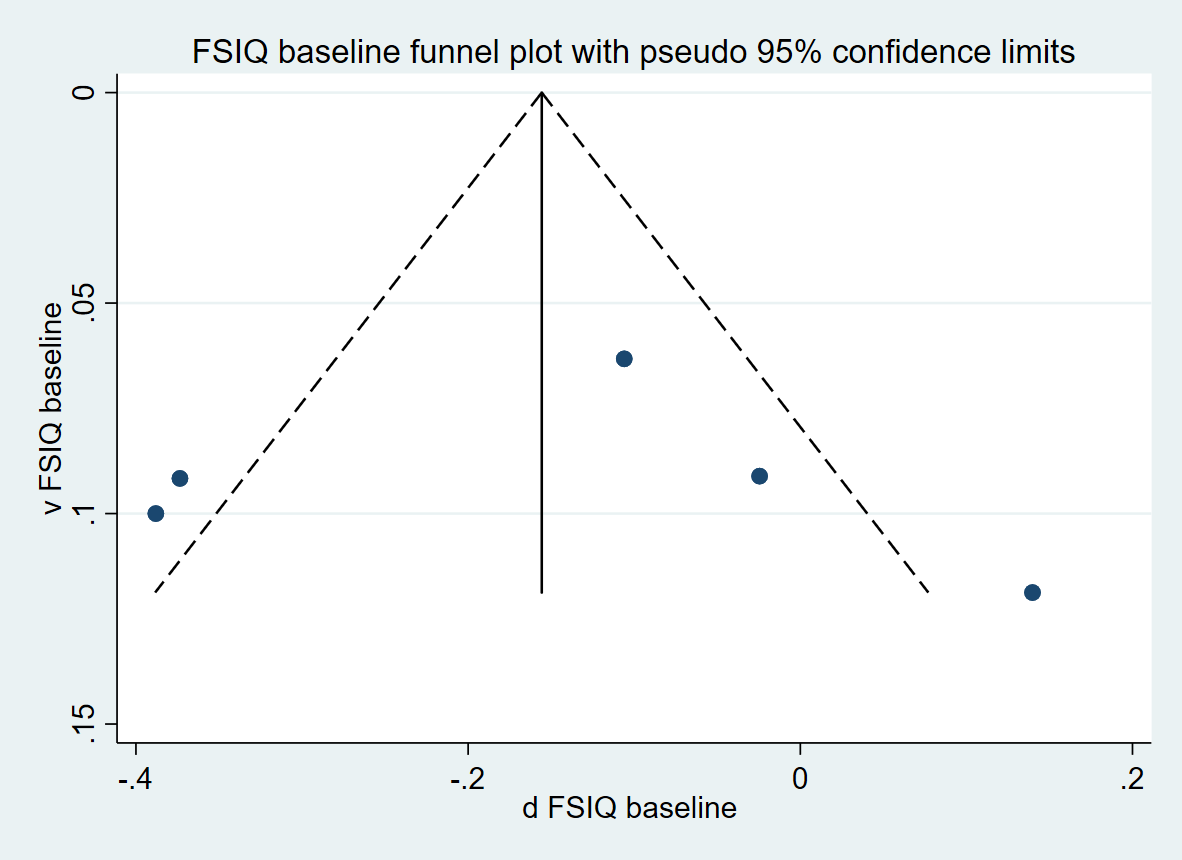


eFigure 9:


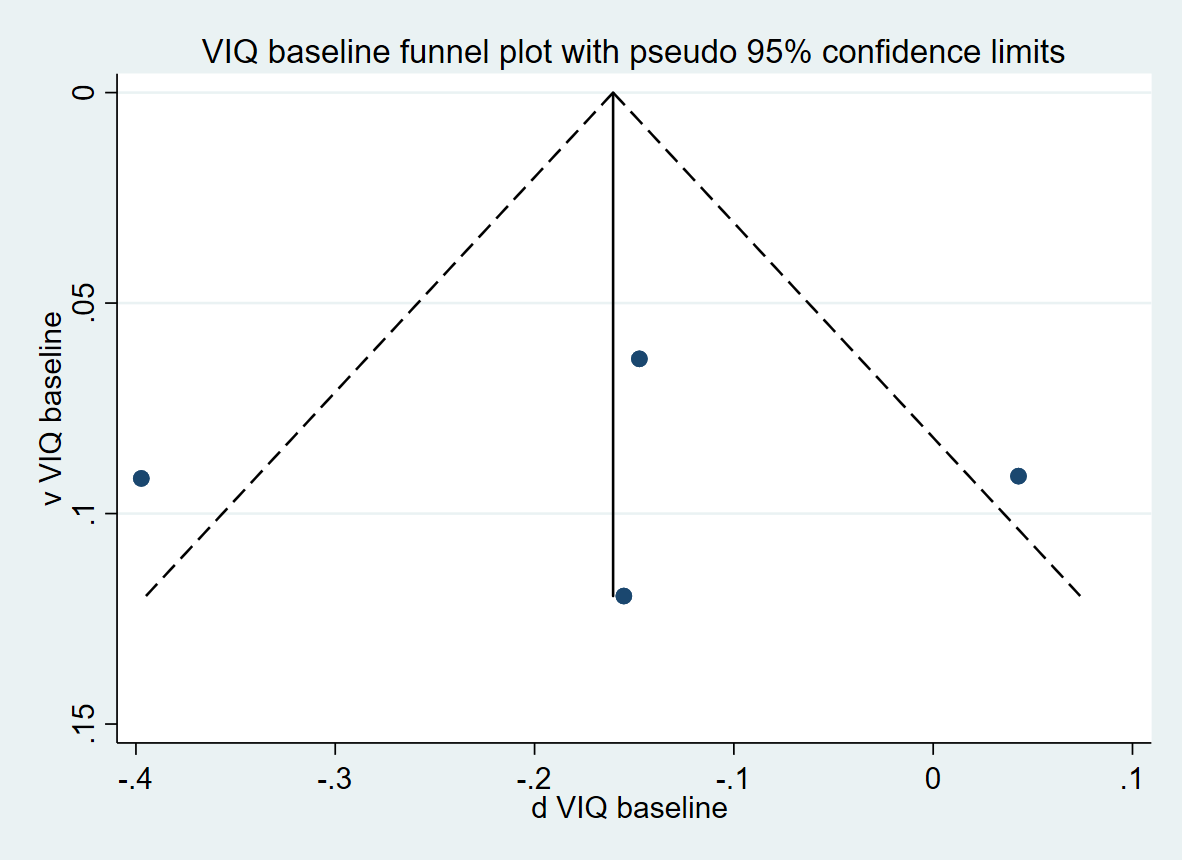


eFigure 10:


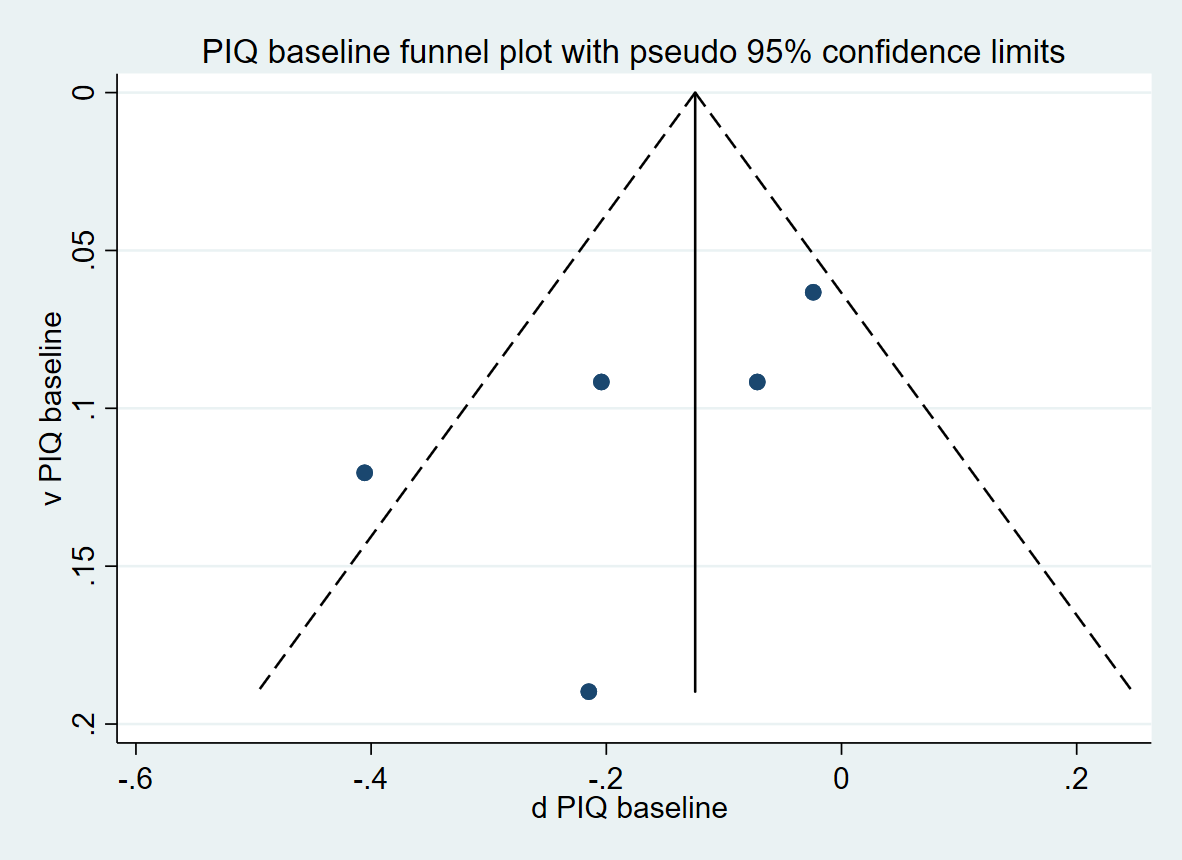


Reference list for studies selected from screening:

Agnew-Blais, J., Seidman, L. J., & Buka, S. L. (2013). Marijuana abuse and dependence and cogntiive decline from childhood to midlife*.* *American Journal of Epidemiology*, *117*(11), S30.

Auer, R., Vittinghoff, E., Yaffe, K., Künzi, A., Kertesz, S. G., Levine, D. A., . . . Sidney, S. (2016). Association between lifetime marijuana use and cognitive function in middle age: the Coronary Artery Risk Development in Young Adults (CARDIA) study. *JAMA Internal Medicine, 176*(3), 352-361.

Bava, S., Jacobus, J., Thayer, R. E., & Tapert, S. F. (2013). Longitudinal changes in white matter integrity among adolescent substance users. *Alcohol Clinical & Experimental Research, 37* (1), e181-189.

Becker, M. P., Collins, P. F., Schultz, A., Urošević, S., Schmaling, B., & Luciana, M. (2018). Longitudinal changes in cognition in young adult cannabis users. *Journal of clinical and experimental neuropsychology, 40*(6), 529-543.

Boccio, C. M., & Beaver, K. M. (2017). Examining the influence of adolescent marijuana use on adult intelligence: Further evidence in the causation versus spuriousness debate. *Drug and alcohol dependence, 177*(1), 199-206.

Brook, J. S., Stimmel, M. A., Zhang, C., & Brook, D. W. (2008). The association between earlier marijuana use and subsequent academic achievement and health problems: A longitudinal study. *American Journal on Addictions, 17*(2), 155-160.

Castellanos-Ryan, N., Pingault, J.-B., Parent, S., Vitaro, F., Tremblay, R. E., & Seguin, J. R. (2017). Adolescent cannabis use, change in neurocognitive function, and high-school graduation: A longitudinal study from early adolescence to young adulthood. *Development and Psychopathology, 29*(4), 1253.

Cengel, H. Y., Bozkurt, M., Evren, C., Umut, G., Keskinkilic, C., & Agachanli, R. (2018). Evaluation of cognitive functions in individuals with synthetic cannabinoid use disorder and comparison to individuals with cannabis use disorder. *Psychiatry Research, 262*, 46-54.

Fried, P., Watkinson, B., & Gray, R. (2005). Neurocognitive consequences of marihuana—a comparison with pre-drug performance. *Neurotoxicology and Teratology, 27*(2), 231-239.

Fried, P., Watkinson, B., James, D., & Gray, R. (2002). Current and former marijuana use: preliminary findings of a longitudinal study of effects on IQ in young adults. *Canadian Medical Association Journal, 166*(7), 887-891.

Friedman, N., Rhee, S., Corley, R., Lessem, J., Stallings, M., Young, S., . . . Hewitt, J. (2014). Marijuana use and impaired cognitive ability*.* *Behavior Genetics*, 44(6), 659.

Hamdi, N., McGue, M., & Iacono, W. (2015). Potential effects of youth cannabis use on adult functioning: a twin study*.* *Behavior Genetics*, *45*,(6), 660.

Ho, B.-C., Barry, A. B., & Koeppel, J. A. (2017). Effects of Marijuana Use on Adolescent Cognitive Development: A Longitudinal Study*.* *Neuropsychopharmacology*, *42*, S629.

Hooper, S. R., Woolley, D., & De Bellis, M. D. (2014). Intellectual, neurocognitive, and academic achievement in abstinent adolescents with cannabis use disorder. *Psychopharmacology, 231*(8), 1467-1477.

Irons, D., Legrand, L., Iacono, W., & McGue, M. (2013). A longitudinal investigation of cannabis use in adolescence and subsequent cognitive decline. *Behavior Genetics*, 43(6), 523.

Jackson, N. J., Isen, J. D., Khoddam, R., Irons, D., Tuvblad, C., Iacono, W. G., . . . Baker, L. A. (2016). Impact of adolescent marijuana use on intelligence: Results from two longitudinal twin studies. *Proceedings of the National Academy of Sciences, 113*(5), E500-E508. doi:10.1073/pnas.1516648113

Kuehn, B. M. (2012). Marijuana use starting in youth linked to IQ loss. *JAMA, 308*(12), 1196. doi:10.1001/2012.jama.12205

Meier, M. H., Caspi, A., Ambler, A., Harrington, H., Houts, R., Keefe, R. S., . . . Moffitt, T. E. (2012). Persistent cannabis users show neuropsychological decline from childhood to midlife. *Proc Natl Acad Sci U S A, 109*(40), E2657-2664. doi:10.1073/pnas.1206820109

Meier, M. H., Caspi, A., Danese, A., Fisher, H. L., Houts, R., Arseneault, L., & Moffitt, T. E. (2018). Associations between adolescent cannabis use and neuropsychological decline: a longitudinal co-twin control study. *Addiction, 113*(2), 257-265. doi:10.1111/add.13946

Meier, M. H., Hill, M. L., Small, P. J., & Luthar, S. S. (2015). Associations of adolescent cannabis use with academic performance and mental health: A longitudinal study of upper middle class youth. *Drug Alcohol Depend, 156*, 207-212. doi:10.1016/j.drugalcdep.2015.09.010

Mendhiratta, S. S., Varma, V. K., Dang, R., Malhotra, A. K., Das, K., & Nehra, R. (1988). Cannabis and cognitive functions: a re-evaluation study. *British Journal of Addiction, 83*(7), 749-753. doi:10.1111/j.1360-0443.1988.tb00506.x

Messinis, L., Kyprianidou, A., Malefaki, S., & Papathanasopoulos, P. (2006). Neuropsychological deficits in long-term frequent cannabis users. *Neurology, 66*(5), 737-739. doi:10.1212/01.wnl.0000201279.83203.c6

Mokrysz, C., Gage, S., Landy, R., Munafo, M., Roiser, J., & Curran, H. (2014). Neuropsychological and educational outcomes related to adolescent cannabis use, a prospective cohort study. *European Neuropsychopharmacology, 24*, S695-696.

Mokrysz, C., Landy, R., Gage, S. H., Munafò, M. R., Roiser, J. P., & Curran, H. V. (2016). Are IQ and educational outcomes in teenagers related to their cannabis use? A prospective cohort study. *Journal of Psychopharmacology, 30*(2), 159-168. doi:10.1177/0269881115622241

Nguyen-Louie, T. T., Castro, N., Matt, G. E., Squeglia, L. M., Brumback, T., & Tapert, S. F. (2015). Effects of Emerging Alcohol and Marijuana Use Behaviors on Adolescents' Neuropsychological Functioning Over Four Years. *Journal of Studies on Alcohol and Drugs, 76*(5), 738-748. doi:10.15288/jsad.2015.76.738

Ross, J., Ellingson, J., Rhee, S., Hewitt, J., Corley, R., & Friedman, N. (2018). The impact of cannabis use on intelligence and executive functioning. *Behavior Genetics, 48,*(6), 511.

Ross, J. M., Ellingson, J. M., Rhee, S. H., Hewitt, J. K., Corley, R. P., Lessem, J. M., & Friedman, N. P. (2020). Investigating the causal effect of cannabis use on cognitive function with a quasi-experimental co-twin design. *Drug and Alcohol Dependence, 206*, e107712. doi:<https://doi.org/10.1016/j.drugalcdep.2019.107712>

Sánchez-Torres, A. M., Basterra, V., Rosa, A., Fañanás, L., Zarzuela, A., Ibáñez, B., . . . Cuesta, M. J. (2013). Lifetime cannabis use and cognition in patients with schizophrenia spectrum disorders and their unaffected siblings. *European Archives of Psychiatry and Clinical Neuroscience, 263*(8), 643-653. doi:10.1007/s00406-013-0404-5

Scheier, L. M., & Botvin, G. J. (1995). Effects of early adolescent drug use on cognitive efficacy in early-late adolescence: a developmental structural model. *Journal of Substance Abuse, 7*(4), 379-404. doi:10.1016/0899-3289(95)90011-x

Tervo-Clemmens, B., Simmonds, D., Calabro, F. J., Montez, D. F., Lekht, J. A., Day, N. L., . . . Luna, B. (2018). Early Cannabis Use and Neurocognitive Risk: A Prospective Functional Neuroimaging Study. *Biological Psychiatry: Cognitive Neuroscience and Neuroimaging, 3*(8), 713-725. doi:10.1016/j.bpsc.2018.05.004

Varma, V. K., Malhotra, A. K., Dang, R., Das, K., & Nehra, R. (1988). Cannabis and cognitive functions: a prospective study. *Drug & Alcohol Dependence, 21*(2), 147-152. doi:10.1016/0376-8716(88)90061-0

Whitmore, C. A., Mikulich-Gilbertson, S., McWilliams, S. K., Corley, R. P., Stallings, M. C., Hewitt, J. K., & Hopfer, C. J. (2018). 2.10 Associations Between Educational Attainment and Marijuana, Tobacco, and Alcohol Use in Early Adulthood in Colorado. *Journal of the American Academy of Child & Adolescent Psychiatry, 57*(10), S162.
